# Tuberculosis testing patterns in South Africa to identify groups that would benefit from increased investigation

**DOI:** 10.1038/s41598-023-47148-y

**Published:** 2023-11-27

**Authors:** Anne N. Shapiro, Lesley Scott, Harry Moultrie, Karen R. Jacobson, Jacob Bor, Abdou M. Fofana, Graeme Dor, Norbert O. Ndjeka, Pedro da Silva, Koleka Mlisana, Helen E. Jenkins, Wendy S. Stevens

**Affiliations:** 1 Department of Biostatistics, Boston University School of Public Health, Boston, USA; 2 Department of Internal Medicine, Faculty of Health Sciences, School of Clinical Medicine, Health Economics and Epidemiology Research Office, University of the Witwatersrand, Johannesburg, South Africa; 3 Wits Diagnostic Innovation Hub, Faculty of Health Sciences, University of the Witwatersrand, Johannesburg, South Africa; 4 Centre for Tuberculosis, A division of the National Health Laboratory Services, National Institute for Communicable Diseases, Johannesburg, South Africa; 5 Division of Infectious Diseases, Boston Medical Center, Boston, USA; 6 Department of Global Health, Boston University School of Public Health, Boston, USA; 7 Questrom School of Business, Institute for Health System Innovation & Policy, Boston University, Boston, USA; 8 National Department of Health, Cape Town, South Africa; 9 National Health Laboratory Service, National Priority Program, Johannesburg, South Africa

**Keywords:** Epidemiology, HIV infections, Tuberculosis

## Abstract

The National Health Laboratory Service (NHLS) collects all public health laboratory test results in South Africa, providing a cohort from which to identify groups, by age, sex, HIV, and viral suppression status, that would benefit from increased tuberculosis (TB) testing. Using NHLS data (2012–2016), we assessed levels and trends over time in TB diagnostic tests performed (count and per capita) and TB test positivity. Estimates were stratified by HIV status, viral suppression, age, sex, and province. We used logistic regression to estimate the odds of testing positive for TB by viral suppression status. Nineteen million TB diagnostic tests were conducted during period 2012–2016. Testing per capita was lower among PLHIV with viral suppression than those with unsuppressed HIV (0.08 vs 0.32) but lowest among people without HIV (0.03). Test positivity was highest among young adults (aged 15–35 years), males of all age groups, and people with unsuppressed HIV. Test positivity was higher for males without laboratory evidence of HIV than those with HIV viral suppression, despite similar individual odds of TB. Our results are an important national baseline characterizing who received TB testing in South Africa. People without evidence of HIV, young adults, and males would benefit from increased TB screening given their lower testing rates and higher test positivity. These high-test positivity groups can be used to guide future expansions of TB screening.

## Introduction

Tuberculosis (TB) is a top ten cause of death globally^1^, yet an estimated 2.9 million individuals with TB disease were undiagnosed in 2019^2^. South Africa is a high burden TB country^1^. TB is the leading cause of death for people living with HIV (PLHIV) in South Africa and nearly 60% of all South African incident TB cases occur in PLHIV^3^. As a result, PLHIV are prioritized for TB screening^4^; however, there are limited data on which populations currently need expanded TB testing efforts.

The South African National Health Laboratory Service’s (NHLS) corporate data warehouse (CDW) houses all results from microbiological TB tests carried out in public sector health laboratories. Linkage of these data into a national laboratory cohort provides an opportunity to capture TB testing patterns stratified by HIV status and, for PLHIV, by viral suppression status (as a proxy for antiretroviral therapy (ART) use)^5,6^. Further stratification by age, sex, and province reveals which groups might benefit from additional TB testing.

Previous work using NHLS data from 2009 to 2011 showed that men received less TB testing than women but did not adjust for age or HIV^7^. South Africa’s recent national TB prevalence survey identified age and sex stratified subgroups with higher TB prevalence and suggested they would benefit from active case finding^8^. The survey stratified by HIV status but not HIV viral suppression, nor did it assess time trends or province-level differences. They found that working aged men not living with HIV to be under-diagnosed with TB. Diagnostic test positivity is another measure of the efficiency of existing case finding in different populations and demonstrates the unmet need for testing^9^. In sub-populations with high test positivity, the benefits of additional testing outreach may be greatest. Here, we analyze national NHLS data from 2012 to 2016 to (1) assess differences in testing frequency by HIV and viral suppression status; (2) determine whether testing patterns changed over the study period, by sex, age, and province; and (3) evaluate test positivity for different sub-populations to assess unmet need for expanded testing. Despite ending in 2016, these data provide the first opportunity to examine trends in TB testing and diagnosis at the national level.

## Methods

### Study context

Prior to redistricting in August 2016, South Africa was divided into 9 provinces, 52 districts, and 234 metropolitan and local municipalities.

GeneXpert MTB/RIF System (Cepheid, Sunnyvale, CA, Xpert) was launched in 2011 and gradually scaled up nationwide with all smear microscopy laboratories having instruments placed by 2013^10^. Studies have shown that Xpert analyser’s implementation increased both the proportion of individuals initiating TB treatment and the proportion who received a microbiological TB diagnostic test^11,12^.

Since 2014, South African policies have recommended testing anyone with TB symptoms and those with TB receive HIV testing^4^. The guidelines state that PLHIV should be TB symptom screened at every health care visit and tested if symptoms present^13^. TB testing for those without symptoms was not recommended.

All PLHIV > 15 years were offered ART if they had CD4 cell counts < 350 (years 2012–2015) or < 500 cells/μl (years 2015–2016); severe or advanced HIV disease, regardless of CD4 cell count; or have active TB disease, are pregnant or breastfeeding, or have known hepatitis B infection^13^.

### National health laboratory service data

We extracted records of all NHLS TB and HIV laboratory tests from January 2012 to December 2016. Data were extracted on July 15, 2017. The NHLS conducts all TB tests in the public sector, accounting for 93% of all TB testing in South Africa^14^. Each TB test record represents a single laboratory TB test which, based on test type, will confirm detection of *Mycobacterium tuberculosis* (*Mtb*) only or detect *Mtb* and provide susceptibility results for anti-TB drug(s). TB laboratory tests included smear microscopy, TB culture by mycobacterial growth indicator tube (MGIT) (Becton, Dickinson, and Company, https://www.bd.com), Xpert MTB/RIF (Cepheid, https://www.cepheid.com) to detect the presence of *Mtb* and rifampicin resistance (RR-TB*)*; MTBDRplus (Hain Lifescience GmbH, https://www.hain-lifescience.de) line probe assays (LPAs) to detect RR-TB and isoniazid resistance; and phenotypic drug susceptibility tests (DST) to detect resistance to isoniazid and second-line drugs (SLD) aminoglycosides (kanamycin) and fluoroquinolones (ofloxacin).

HIV diagnosis is typically determined by rapid test and not captured in the laboratory database. Once diagnosed, PLHIV have a baseline CD4 count measuring levels of T lymphocytes (CD4) in the blood. Additionally, patients who start ART have regular HIV viral load (VL) tests to monitor for treatment impact^5,6^. We therefore used the presence of CD4 count or VL results as an indicator for whether the patient had HIV.

NHLS data include a patient identifier, but its inconsistent use and manual capture introduces errors. To track individuals over time, a person matching algorithm, previously applied to NHLS HIV^5,6^ and TB data^15,16^ was implemented to link samples belonging to the same individual. The algorithm estimates the probability that records belong to the same individual based on name, birthdate, sex, and facility. Validation of HIV (TB) linkage indicated that the algorithm correctly identifies 94% (92%) of true matches and 99% (91%) of the identified matches were correctly assigned^16^; lower performance of the linkage for TB labs was attributed in part to the larger underlying population of patients screened for TB.

### Other data sources

Total, HIV and ART population estimates by age, sex, and province were obtained from the Thembisa model^17^. The population of people without HIV was defined as the HIV infected population subtracted from the total population. The population of those not on ART was defined as the ART population subtracted from the HIV population. We assume the population on ART were virally suppressed, given that most individuals who are not virally suppressed are not on ART.

### Definitions

A person with confirmed TB is someone with at least one positive TB test (smear, culture and/or Xpert). To define an episode of TB disease (henceforth, “TB episode”), we grouped a person’s TB tests over a one- or two-year period following the taken date of the individual’s first confirmed positive TB test if they had rifampicin-susceptible or RR-TB respectively. RR-TB was defined by at least one test with rifampicin resistance on Xpert MTB/RIF or LPA tests within 14 days of first positive TB result. One individual could have multiple TB episodes.

We defined a PLHIV as someone with at least one HIV VL or CD4 cell count test and the HIV diagnosis date as the date of the first VL or CD4 test. We defined an individual as having no laboratory evidence of HIV (henceforth, “people without HIV”) if they had no recorded VL or CD4 tests.

We defined PLHIV as virally suppressed if their VL $\le$ 1000 copies per milliliter of blood and the date of viral suppression as the first date the individual’s VL was $\le$ 1000^18^. If a suppressed individual subsequently has a VL above 1000, we then consider them PLHIV not virally suppressed as of the date of that VL test. If the individual’s VL subsequently goes below 1000, we consider them suppressed again. Although current definitions of viral suppression use lower thresholds such as 50 copies per ml, we use this higher threshold to enable comparisons across study periods and geographic areas that used viral load testing methods with different levels of sensitivity.

We defined a TB test as belonging to a PLHIV if they were diagnosed with HIV before or up to 1 year after the TB test date. As most individuals’ initial HIV test either precedes or occurs on the same day as an individuals’ first TB diagnosis (Appendix Fig. A1), we believe misclassification of HIV status at the time of TB diagnosis is low. We defined a TB test as belonging to someone virally suppressed if the test was performed during a period in which they were considered suppressed.

### Test inclusions and definitions

*Exclusions* test records with a facility that could not be linked to a province, and all tests associated with a TB episode containing no positive TB tests (e.g., because the episode began before 2012 and all of the episode’s positive tests were in an earlier year or none of the episode’s positive tests could be linked to a province). See appendix Fig. S2 for cohort definition flow diagram.

A diagnostic TB test was one used to determine if someone had TB rather than those carried out to monitor treatment response. We considered diagnostic tests within a two-week period as part of the same diagnostic evaluation and not as separate tests; we refer to these groupings as TB testing periods henceforth. We grouped tests done subsequently within a TB episode into four-week periods, such that all tests in a four-week period would count as one TB follow up evaluation, as we expect individuals to be tested at most monthly during their TB treatment. We consider these tests as monitoring tests. All analyses presented in the main text include only diagnostic tests; see table S1 for analyses including non-diagnostic tests.

### Analysis

#### TB testing measures

We stratified analyses by HIV and viral suppression status. We calculated multiple measures to examine TB testing patterns: absolute number of testing periods, testing periods per capita (the absolute number of tests divided by the relevant population), the number of positive testing periods (testing periods with at least one positive TB test), test positivity (the number of positive testing periods divided by the absolute number of testing periods), number of testing periods per TB episode (the number of testing periods associated with a TB episode divided by the number of people with TB and number of TB episodes, respectively), and the mean number of unique diagnostic test types received. Individuals could receive a maximum of seven unique diagnostic tests: culture, DST1 (DST for first line drugs; included drugs varied by province), DSTX (DST extended; fluoroquinolone and second line injectables), Xpert, LPA, PCR, or smear. Since Xpert implementation, most smear tests are follow-ups per WHO guidelines. We estimated these measures both over the entire study period (2012–2016) and population and stratified by year and province to examine testing trends temporally and spatially.

We stratified test positivity by age and sex to identify specific demographic groups that might benefit from additional testing, assuming higher test positivity indicated increased under-detection, excluding test records missing age and sex. We also calculated the proportion of all TB tests that occurred within each age/sex stratum of each HIV status/viral suppression group and the proportion of the population in each group. If a person had tests performed in multiple provinces within one year, they were assigned to the province in which they had the most tests.

#### Individual odds of testing positive

We used logistic regression to determine the odds of an individual having a positive TB test result (conditional on receiving a TB test) for (a) PLHIV not virally suppressed, and (b) virally suppressed PLHIV, each compared with (c) people without HIV. For each group, we focused on the six-month period immediately after HIV diagnosis, start of viral suppression, or receiving a test for something other than HIV or TB, respectively. Note that the reference group (c) includes only those who are not diagnosed with HIV throughout the entire study period. We excluded individuals who switched between any of these groups during the six-month period (e.g. those who became suppressed less than six months after their HIV diagnosis or diagnosed with HIV less than six months after their test for something else) or whose initial HIV diagnosis, first date of viral suppression, or first test for something other than HIV or TB was after July 1, 2016 to ensure consistent follow-up time for each group. We adjusted for sex and age and excluded anyone missing age or sex.

We used R 4.1.0 for data analysis.

### Ethics

Ethics approval was obtained from the Boston University Medical Campus IRB (H-31968) and, University of Witwatersrand Human Research Ethics Committee (M160978). The National Health Laboratory Service AARMS review team approved sharing of these data (PR2223709). This study was considered no greater than minimal risk by the IRB. Given the practicalities of retrospectively contacting everyone in this large laboratory database, waiver of consent was considered appropriate by the IRB. All methods were carried out in accordance with relevant guidelines and regulations.

## Results

### Population characteristics of our cohort

A total of 2,461,718 (6.65%) records were excluded due to missing facility or being associated with a TB episode containing no positive tests. Of the people in our study, 3,220,565 (35%) were laboratory confirmed to be PLHIV and of those, 649,353 (20%) were virally suppressed at some point in the study period. Among PLHIV, females accounted for 63% and 59% of those virally suppressed and those not virally suppressed respectively (Table 1).

**Table 1:** Characteristics of the cohort that received at least one TB test during 2012–2016 stratified by laboratory confirmed HIV and viral suppression statuses.

		People without HIV		PLHIV virally suppressed		PLHIV not virally suppressed	
		Number*	Proportion	Number*	Proportion	Number*	Proportion
	Total	5,880,960		649,353		2,571,212	
Sex	Female	2,967,148	0.505	408,472	0.629	1,507,307	0.586
	Male	2,772,276	0.471	236,136	0.364	1,037,465	0.403
	Unknown	141,536	0.024	4,745	0.007	26,440	0.010
Age (years)	< 19	1,163,709	0.189	32,341	0.050	148,100	0.058
	20–34	1,473,052	0.261	221,929	0.342	1,055,294	0.410
	35–49	1,086,851	0.200	271,951	0.419	959,041	0.373
	50–64	973,209	0.158	99,549	0.153	309,157	0.120
	> 65	560,112	0.087	12,401	0.019	39,650	0.015
	Unknown	624,027	0.104	11,182	0.017	59,970	0.023
Province**	EC	1,174,465	0.200	88,984	0.137	318,071	0.124
	FS	277,981	0.047	43,002	0.066	161,749	0.063
	GP	782,702	0.133	102,845	0.158	589,073	0.229
	KZN	1,599,012	0.272	217,257	0.335	726,979	0.283
	LP	611,988	0.104	51,073	0.079	193,249	0.075
	MP	278,272	0.047	44,923	0.069	187,658	0.073
	NC	170,738	0.029	12,138	0.019	46,977	0.018
	NW	350,752	0.060	43,013	0.066	189,975	0.074
	WC	635,050	0.108	46,118	0.071	157,481	0.061
TB status	No TB episodes in study period	5,311,696	0.903	496,904	0.765	2,120,573	0.825
	Had at least 1 TB episode	569,264	0.097	152,449	0.235	450,639	0.175
Rifampin resistance status***	No evidence of RR-TB	405,566	0.712	108,316	0.711	346,253	0.768
	At least 1 RR-TB episode	28,532	0.050	17,989	0.118	25,456	0.056
	Was not tested for RR TB	135,166	0.237	26,144	0.171	78,930	0.175

*Number of unique individuals in the NHLS data.

**EC: Eastern Cape, FS: Free State, GP: Gauteng, KZN: KwaZulu-Natal, LP: Limpopo, MP: Mpumalanga, NC: Northern Cape, NW: North West, WC: Western Cape.

***Amongst individuals with at least one TB episode.

### National level TB testing patterns stratified by HIV and viral suppression statuses

A total of 13,347,295 diagnostic TB testing periods were carried out during the study period. Of these, 4,654,275 testing periods (35%) were among PLHIV without documented viral suppression and 1,019,397 (8%) were among virally suppressed PLHIV, resulting in 0.32 diagnostic testing episodes per capita among PLHIV without documented viral suppression and 0.08 among PLHIV with viral suppression, compared to 0.03 in the population without HIV (Table 2). Diagnostic TB test positivity was higher among PLHIV without viral suppression (12%) compared to PLHIV who were virally suppressed (8%) and people without HIV (8%). Calculations using all testing episodes showed similar trends (Appendix Table A1). Virally suppressed PLHIV received a greater average number of unique types of diagnostic TB tests (2.23) than PLHIV not virally suppressed (2.03) and those without HIV (1.88) (Table 2).

**Table 2:** Tuberculosis (TB) testing measures amongst individuals in the NHLS data stratified by laboratory confirmed HIV and viral suppression statuses.

	People without HIV	PLHIV virally suppressed	PLHIV not virally suppressed
Number of diagnostic TB testing periods	7,673,623	1,019,397	4,654,275
TB diagnostic testing periods per capita*	0.03	0.08	0.32
Diagnostic TB test positivity**	7.91%	7.83%	11.86%
Number of TB episodes	603,098	131,600	508,852
Number of people with laboratory diagnosed TB	574,563	123,777	474,012
Number of people with laboratory diagnosed TB per 100,000 population*	240	960	3220
Mean number of unique diagnostic test types***	1.88	2.32	2.03

*Population denominators are Thembisa estimates.

**Denominators from NHLS data.

***Individuals could receive a maximum of 7 unique diagnostic tests: culture, DST1, DSTX, Xpert, LPA, PCR, or smear.

### Province-level TB testing patterns over time

**Figure 1:**
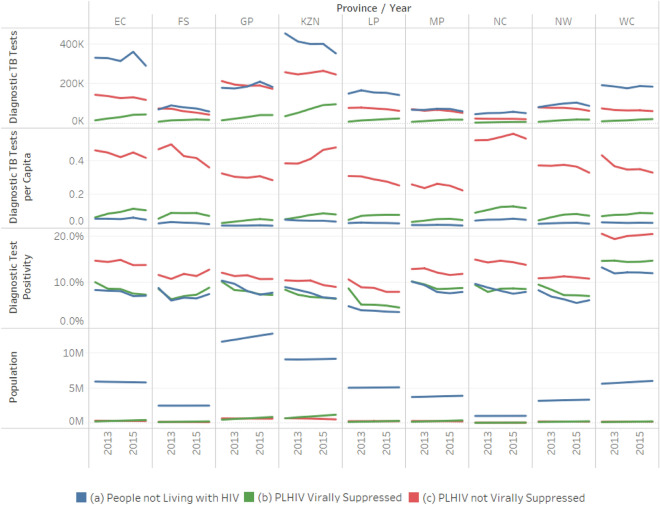
TB testing trends by HIV and viral suppression status, province, and year.

KwaZulu-Natal, Gauteng, and Eastern Cape consistently carried out the most diagnostic tests annually (Fig. 1, first row). We observed a general trend of decreasing testing annually across provinces for the total population. More diagnostic TB tests were carried out among PLHIV not virally suppressed than those virally suppressed (Fig. 1). However, the number performed on virally suppressed PLHIV increased over time, while the number among PLHIV not virally suppressed decreased, likely reflecting increasing numbers of PLHIV initiating ART.

Diagnostic TB tests per capita was higher among PLHIV not virally suppressed in all provinces (Fig. 1, second row). Testing per capita decreased in all groups and provinces over time. Test positivity decreased sharply from 2012 to 2013 and then plateaued in all provinces (Fig. 1, third row). Test positivity was consistently higher among PLHIV not virally suppressed than people without HIV and virally suppressed PLHIV over time and across provinces.

### Individual-level odds of testing positive for TB

Of those that received TB tests, PLHIV not virally suppressed were more likely to test positive for TB than people without HIV (OR = 1.49, [95% CI 1.47, 1.50]) after adjusting for age and sex (Table 3). The odds of a positive TB test were similar for virally suppressed PLHIV as for people without HIV (OR = 1.08, [95% CI 1.05, 1.12]) (Table 4). Males were more likely to test positive than females in both analyses (OR = 1.60, [95% CI 1.57, 1.63] comparing PLHIV with viral suppression to no HIV, OR = 1.79, [95% CI 1.77, 1.81] comparing PLHIV not yet virally suppressed to no HIV).

**Table 3:** Odds of having a positive TB test in the NHLS data (conditional on receiving a TB test) comparing people in the first six months after a HIV test (pre viral suppression) and another laboratory test for those not living with HIV.

	Received positive TB test result		Odds ratio adjusted for age and sex (95% CI)
	Yes	No	
Without HIV	59,842	422,797	Ref
Pre viral suppression	172,282	845,582	1.49 (1.47, 1.50)
Female	90,182	673,259	Ref
Male	141,942	595,120	1.79 (1.77, 1.81)
Age (years)			1.01 (1.01, 1.01)

**Table 4:** Odds of having a positive TB test in the NHLS data (conditional on receiving a TB test) comparing people in the first six months after becoming virally suppressed (post viral suppression) and another laboratory test for those not living with HIV.

	Received positive TB test result		Odds ratio adjusted for age and sex (95% CI)
	Yes	No	
Without HIV	59,842	422,797	Ref
Post viral suppression	6195	41,425	1.08 (1.05, 1.12)
Female	25,077	230,177	Ref
Male	40,960	234,045	1.60 (1.57, 1.63)
Age (years)			1.01 (1.01, 1.01)

### Test positivity by age and sex

**Figure 2:**
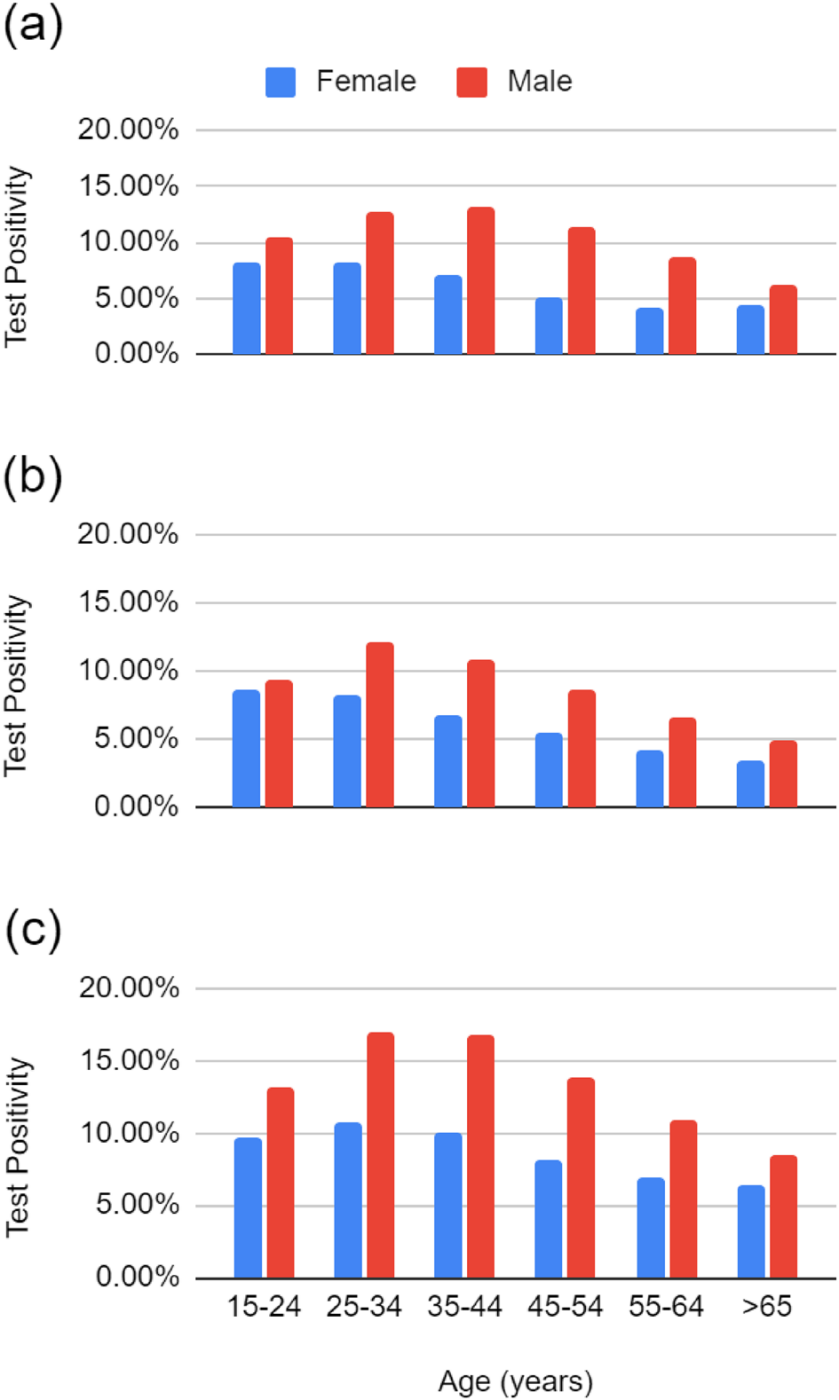
Test positivity of diagnostic TB tests for (**a**) people with no laboratory evidence of HIV, (**b**) virally suppressed PLHIV, and (**c**) PLHIV not virally suppressed.

TB test positivity varied substantially by age, sex, and HIV and viral suppression status (Fig. 2). Test positivity was higher among males compared to females and individuals aged 15–44, as well as males without HIV than among virally suppressed males living with HIV. This trend held constant over time and across provinces (Appendix Tables A3–A18). Stratifying by age, test positivity was higher for people aged 15–34 years old and males aged 35–44 than older age groups. Among people aged 15–24 years, both males and females without documented HIV made up a higher proportion of those receiving TB tests than of the population and males made up a lower proportion of those receiving TB tests than of the population. This trend held for PLHIV not virally suppressed but reversed for virally suppressed PLHIV (Appendix Table A2).

## Discussion

Between 2012 and 2016, PLHIV received substantially more TB tests per capita than people without HIV in the public sector. PLHIV who were not virally suppressed had higher TB test positivity, consistent with TB being an opportunistic infection associated with HIV. Once PLHIV were on treatment and virally suppressed, their TB test positivity was similar to people without HIV. Regardless of HIV and viral suppression statuses, males had higher test positivity than females, as did people aged 15–44 years compared with other age groups. These high test positivity groups suggest that working age males were the most under-diagnosed demographic in the study period.

We observe decreasing testing across all provinces during our study period, which corresponds to a decline in TB incidence in the same period. People are only tested for TB if they receive a positive symptom screen, as such with lowered TB incidence we would expect to see fewer TB tests performed. This declining TB incidence is also seen in the declining TB test positivity during our study period. This decline in TB incidence has been attributed to increased ART coverage and scaled-up efforts to identify TB cases between 2005 and 2012^19,20^.

A 2016 systematic review found that although TB incidence rates are generally higher among males than females, males are less likely to access care^21^. Our results are consistent with this and other studies in South Africa that found that males were less frequently tested for TB compared with females of the same age^7,8^. Several contributory factors exist such as social pressure to ignore symptoms or illness^22^, and stigma^23^. Conversely, women are more likely to attend health facilities for reproductive health services, increasing their chances of TB testing. Since undiagnosed TB is likely a major driver of transmission, finding innovative ways to increase testing among men will be critical to reducing TB in South Africa.

People aged 15–44 years—males and females—had higher test positivity than other age groups in their respective sex strata, indicating particular under-diagnosis in this age group. This is consistent with other studies^24,25^ and may in part be due to working aged people being less able to access health services due to being at work when those services are open^26^. Again, innovative active case-finding methods, for example, placing mobile diagnostic services at workplaces or transport terminals^27^, may help increase TB testing among this age group.

The recent South African TB Prevalence Survey found that TB was particularly under-detected among people without HIV and suggested this is a group that needs more testing^8^. Our results support this conclusion and demonstrate their consistency across time and geographic location. PLHIV who were not virally suppressed had higher test positivity than virally suppressed PLHIV, reflecting the known biology that PLHIV not on ART are at high TB disease risk^28–30^. However, virally suppressed PLHIV had a similar risk of developing TB to those without HIV. When considered in tandem with test positivity, males without HIV were particularly at risk of not receiving testing. This difference was not apparent among females. Identifying opportunities for active case-finding among males is complex, and even harder among those without HIV as they interact less with healthcare services and may only seek care when TB symptoms are severe. PLHIV are understandably prioritized for TB testing, but our study re-enforces the need for strategies for more active case finding among the HIV uninfected population, especially males.

Systematic Targeted Universal Testing for Tuberculosis (TUTT), implemented as a randomized trial from 2018 to 2020, found a 17% increase in TB diagnoses per month compared to the prior year after implementing routine TB testing for individuals with documented HIV infections, close contacts, or diagnosed TB in the prior two years^31^. The South African TB Prevalence Survey found that 57.7% of people with bacteriologically confirmed TB initially screened positive with chest x-ray alone^8^, without symptoms. Incorporating chest x-ray, which is not currently part of the TB screening algorithm^4^, more often, particularly targeted towards specific groups, could help close some of the testing gaps we have observed.

A study strength is the use of national TB testing and HIV data, consisting of around 25 million TB tests over five years allowing us to stratify by age, sex, province, and HIV and viral suppression statuses. This is a critical tool for understanding who is receiving TB bacteriological testing across South Africa.

Limitations include the absence of individual identifiers and resulting possible false matches from the record linkage algorithm. However, the algorithm has been rigorously validated and has high sensitivity and specificity^15,16^ and thus errors are unlikely to have systematically biased our results. Also, these data include only laboratory tests from those who sought care and could produce a sputum sample. Many PLHIV cannot produce a sputum sample^32^. Therefore, people diagnosed with TB based on clinical signs and symptoms alone, and anyone with undiagnosed TB would not be included in our data. Furthermore, only individuals who have a positive symptom screen are tested for TB, and symptom screening has a known low sensitivity^33^. PLHIV beginning ART also engage with care at a higher frequency, were more likely to receive a TB test, and more likely to receive multiple different TB tests, increasing the chances of testing positive while also lowering test positivity. Using testing periods rather than individual tests mitigates some of this bias, but PLHIV are still receiving more tests and thus have more opportunities to test positive for TB and have a positive testing period.

It is possible that the nationwide rollout of Xpert during 2012 influenced our results. However, results excluding 2012 (Appendix Table A19) show similar trends to results including 2012 (Table 1). Finally, we consider two possible sources of misclassification. We did not have laboratory confirmation of absence of HIV infection, which could introduce misclassification. Additionally, we note the seemingly low percentage (20%) of virally suppressed PLHIV in our study compared to the Thembisa model’s estimate (43%)^17^. This may be due to some individuals lacking a viral load test to confirm viral suppression and thus not being classified as such and may be further exacerbated by occasional blips in viral load measurements that can occur due to a variety of reasons.

Finally, we acknowledge that these data end in 2016. The linkage algorithm used on these data is currently being implemented into routine data flows, but its use at such scale has proved challenging. In the interim, these data are providing critical guidance. Policy changes since 2016, such as TUTT^31^ and Universal Test and Treat^34^, and the call for increased screening for TB during pregnancy^35^ continue to focus resources towards women and PLHIV, further exacerbating the trends we show.

Our data provide a baseline of the TB testing situation in South Africa. Our finding that intensified case finding is needed for people not living with HIV, males, and young adults can be used to guide in active case finding and should be a critical consideration as South Africa continues to advance its TB testing program.

### Supplementary Information

Supplementary Information.

## Data Availability

Access to primary data is subject to restrictions owing to privacy and ethics policies set by the South African Government. Requests for access to the data can be made via the Office of Academic Affairs and Research at the National Health Laboratory Service through the AARMS research project application portal: https://aarms.nhls.ac.za/.
