# Supplementary material for: Tuberculosis testing patterns in South Africa to identify groups that would benefit from increased investigation
Source: Sci Rep. 2023 Nov 27;13:20875. doi: 10.1038/s41598-023-47148-y (PMC10682361; doi:10.1038/s41598-023-47148-y)
Supplement: Supplementary file 1 — Supplementary Information. [file 41598_2023_47148_MOESM1_ESM.docx]

**Tuberculosis testing patterns in South Africa to identify groups that would benefit from increased investigation: Supplementary Appendix**

Contents

Figure A1 - Time from first TB diagnosis till first HIV test……………………………………………………………………………………………………………………………………………………………….2

[Figure A2 – Cohort defining flow diagram 3](#_Toc115194940)

[Table A1 – TB testing measures, including monitoring tests 4](#_Toc115194941)

[Table A2 – Test positivity by HIV and viral suppression status, age and sex 5](#_Toc115194941)

[Table A3 — Test positivity by HIV and viral suppression status and year 6](#_Toc115194942)

[Table A4 — Test positivity by HIV and viral suppression status, age, and sex for 2012 7](#_Toc115194943)

[Table A5 — Test positivity by HIV and viral suppression status, age, and sex for 2013 8](#_Toc115194944)

[Table A6 — Test positivity by HIV and viral suppression status, age, and sex for 2014 9](#_Toc115194945)

[Table A7 — Test positivity by HIV and viral suppression status, age, and sex for 2015 10](#_Toc115194946)

[Table A8 — Test positivity by HIV and viral suppression status, age, and sex for 2016 11](#_Toc115194947)

[Table A9 — Test positivity by HIV and viral suppression status and province 12](#_Toc115194948)

[Table A10 — Test positivity by HIV and viral suppression status, age, and sex for EC 13](#_Toc115194949)

[Table A11 — Test positivity by HIV and viral suppression status, age, and sex for FS 14](#_Toc115194950)

[Table A12 — Test positivity by HIV and viral suppression status, age, and sex for GP 15](#_Toc115194951)

[Table A13 — Test positivity by HIV and viral suppression status, age, and sex for KZN 16](#_Toc115194952)

[Table A14 — Test positivity by HIV and viral suppression status, age, and sex for LP 17](#_Toc115194953)

[Table A15 — Test positivity by HIV and viral suppression status, age, and sex for MP 18](#_Toc115194954)

[Table A16 — Test positivity by HIV and viral suppression status, age, and sex for NC 19](#_Toc115194955)

[Table A17 — Test positivity by HIV and viral suppression status, age, and sex for NW 20](#_Toc115194956)

[Table A18 — Test positivity by HIV and viral suppression status, age, and sex for WC 21](#_Toc115194957)

[Table A19 — TB testing measures, excluding 2012 data 22](#_Toc115194958)

Figure A1 Time (days) from 1^st^ TB diagnosis till 1^st^ HIV test. Negative time indicates that HIV test preceded TB diagnosis. Note we have HIV tests outside of study period (1986-2019).


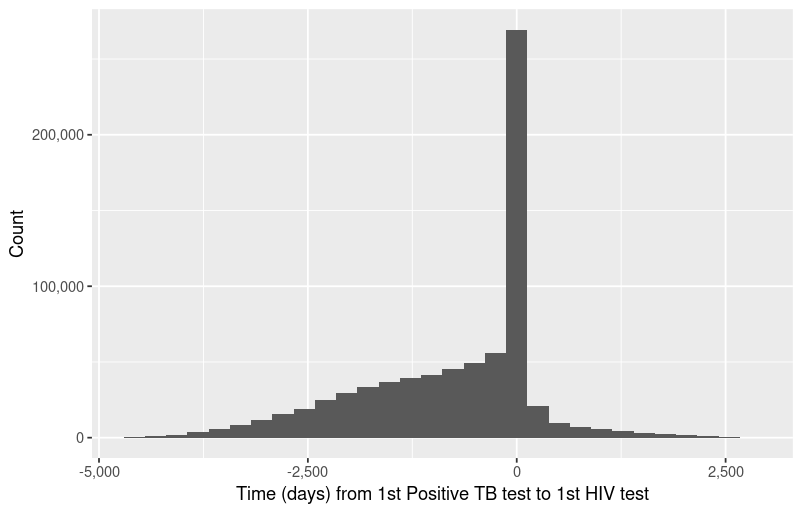


Figure A2 Flow chart defining National Health Laboratory Service (NHLS) cohort


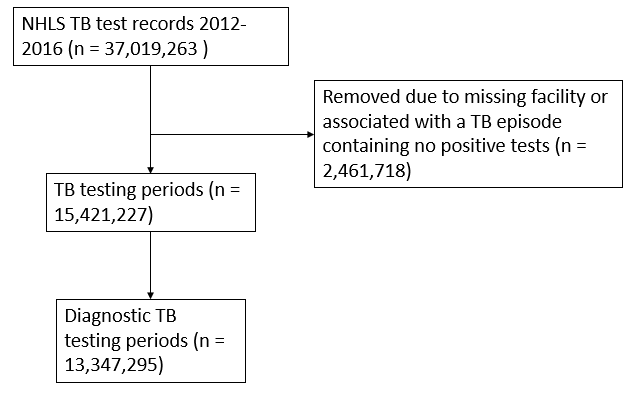


# Table A1 Tuberculosis (TB) testing measures amongst individuals in the NHLS data stratified by laboratory confirmed HIV and viral suppression statuses, including both diagnostic and monitoring tests.

|  | People without HIV | PLHIV virally suppressed | PLHIV not virally suppressed |
| --- | --- | --- | --- |
| Number of TB testing periods | 8,492,693 | 1,343,329 | 5,585,205 |
| TB testing periods per capita* | 0.04 | 0.10 | 0.38 |
| TB testing periods per TB episode** | 2.37 | 3.07 | 2.92 |
| TB test positivity** | 9.25% | 10.20% | 13.23% |
| Number of positive TB testing periods | 785,887 | 136,999 | 739,023 |

*Population denominators are Thembisa estimates

**Denominators from NHLS data

***Individuals could receive a maximum of 7 unique diagnostic tests: culture, DST1 (DST for first line drugs), DSTX (DST extended), Xpert, LPA, PCR, or smear

Table A2 Test positivity of diagnostic TB tests stratified by HIV and viral suppression status, and age and sex. Test positivity is shaded to reflect the magnitude of the value; higher test positivity is shaded darker green. Proportion of number tested and proportion of population are shaded if the proportion of the population exceeds the proportion of number tested, as this could indicate populations that are currently under-tested for TB.

|  |  | People without HIV | | | | PLHIV virally suppressed | | | | PLHIV not virally suppressed | | | |
| --- | --- | --- | --- | --- | --- | --- | --- | --- | --- | --- | --- | --- | --- |
| **Age (years)** | **Sex** | **Test positivity** | **Number tested** | **Proportion of number tested*** | **Proportion of population**** | **Test positivity** | **Number tested** | **Proportion of number tested*** | **Proportion of population**** | **Test positivity** | **Number tested** | **Proportion of number tested*** | **Proportion of population**** |
| 15-24 | F | 8.2% | 485,211 | 10.4% | 14.2% | 8.7% | 29,727 | 4.9% | 3.9% | 9.6% | 245,927 | 8.6% | 14.8% |
| 15-24 | M | 10.4% | 420,035 | 9.0% | 15.3% | 9.4% | 6,771 | 1.1% | 1.0% | 13.1% | 64,770 | 2.3% | 16.2% |
| 25-34 | F | 8.2% | 491,289 | 10.5% | 10.2% | 8.3% | 131,699 | 21.5% | 28.9% | 10.8% | 624,234 | 21.9% | 9.0% |
| 25-34 | M | 12.8% | 535,009 | 11.4% | 13.0% | 12.1% | 45,332 | 7.4% | 7.9% | 17.0% | 354,480 | 12.4% | 13.3% |
| 35-44 | F | 7.0% | 373,868 | 8.0% | 8.0% | 6.7% | 124,068 | 20.2% | 23.4% | 10.0% | 459,564 | 16.1% | 7.0% |
| 35-44 | M | 13.2% | 408,647 | 8.7% | 8.2% | 10.9% | 80,291 | 13.1% | 11.8% | 16.7% | 406,754 | 14.2% | 8.0% |
| 45-54 | F | 5.1% | 382,452 | 8.2% | 7.3% | 5.4% | 71,588 | 11.7% | 10.7% | 8.1% | 246,421 | 8.6% | 7.0% |
| 45-54 | M | 11.4% | 355,908 | 7.6% | 6.2% | 8.6% | 53,961 | 8.8% | 6.2% | 13.9% | 220,390 | 7.7% | 6.2% |
| 55-64 | F | 4.2% | 341,169 | 7.3% | 5.2% | 4.2% | 30,350 | 5.0% | 3.3% | 6.9% | 95,244 | 3.3% | 5.3% |
| 55-64 | M | 8.6% | 307,739 | 6.6% | 4.1% | 6.6% | 24,210 | 4.0% | 2.1% | 10.9% | 88,511 | 3.1% | 4.2% |
| >65 | F | 4.3% | 329,460 | 7.0% | 5.2% | 3.5% | 8,246 | 1.3% | 0.5% | 6.4% | 25,519 | 0.9% | 5.5% |
| >65 | M | 6.1% | 249,465 | 5.3% | 3.2% | 5.0% | 6,590 | 1.1% | 0.4% | 8.5% | 23,557 | 0.8% | 3.4% |
| Total |  | 8.6% | 4,680,252 | 58.9% | 86.2% | 8.0% | 612,833 | 7.3% | 8.4% | 12.1% | 2,855,371 | 33.8% | 5.4% |

*Numerator (number in subpopulation who received a test) and denominator (total number in population in the cohort) taken from NHLS data

** Numerator (number estimated in subpopulation) and denominator (total estimated population) taken from Thembisa 2012 estimates

Table A3 Test positivity of diagnostic TB tests stratified by HIV and viral suppression status and year.

|  | People not living with HIV | | | | PLHIV virally suppressed | | | | PLHIV not virally suppressed | | | |
| --- | --- | --- | --- | --- | --- | --- | --- | --- | --- | --- | --- | --- |
| **Year** | **Test positivity** | **Number tested** | **Proportion of number tested*** | **Proportion of population**** | **Test positivity** | **Number tested** | **Proportion of number tested*** | **Proportion of population**** | **Test positivity** | **Number tested** | **Proportion of number tested*** | **Proportion of population**** |
| 2012 | 9.8% | 1,056,011 | 0.567 | 0.875 | 9.8% | 70820 | 0.038 | 0.053 | 12.7% | 743978 | 0.396 | 0.072 |
| 2013 | 8.9% | 1,062,240 | 0.566 | 0.866 | 8.2% | 114358 | 0.06 | 0.065 | 12.1% | 707042 | 0.373 | 0.069 |
| 2014 | 8.4% | 1,067,192 | 0.558 | 0.86 | 7.9% | 156513 | 0.081 | 0.074 | 12.3% | 696600 | 0.361 | 0.066 |
| 2015 | 7.8% | 1,140,053 | 0.561 | 0.853 | 7.6% | 201674 | 0.098 | 0.084 | 11.6% | 700838 | 0.341 | 0.063 |
| 2016 | 7.9% | 1,012,865 | 0.547 | 0.847 | 7.6% | 212181 | 0.114 | 0.093 | 11.6% | 634154 | 0.34 | 0.06 |

*Numerator (number in subpopulation who received a test) and denominator (total number in population in the cohort) taken from NHLS data

** Numerator (number estimated in subpopulation) and denominator (total estimated population) taken from Thembisa 2012 estimates

Table A4 Test positivity of diagnostic TB tests stratified by HIV and viral suppression status, sex, and age for year 2012.

|  |  |  | People not living with HIV | | | | PLHIV virally suppressed | | | | PLHIV not virally suppressed | | | |
| --- | --- | --- | --- | --- | --- | --- | --- | --- | --- | --- | --- | --- | --- | --- |
| **Year** | **Age (years)** | **Sex** | **Test positivity** | **Number tested** | **Proportion of number tested*** | **Proportion of population**** | **Test positivity** | **Number tested** | **Proportion of number tested*** | **Proportion of population**** | **Test positivity** | **Number tested** | **Proportion of number tested*** | **Proportion of population**** |
| 2012 | 15-24 | F | 9.3% | 112582 | 0.107 | 0.142 | 11.2% | 3069 | 0.043 | 0.039 | 10.6% | 64848 | 0.087 | 0.135 |
| 2012 | 15-24 | M | 11.7% | 88190 | 0.084 | 0.153 | 12.2% | 662 | 0.009 | 0.01 | 15.0% | 13468 | 0.018 | 0.025 |
| 2012 | 25-34 | F | 9.3% | 119339 | 0.113 | 0.102 | 10.0% | 15721 | 0.222 | 0.289 | 11.4% | 175463 | 0.236 | 0.292 |
| 2012 | 25-34 | M | 15.1% | 110261 | 0.104 | 0.13 | 14.2% | 5385 | 0.076 | 0.079 | 18.1% | 84421 | 0.113 | 0.15 |
| 2012 | 35-44 | F | 8.2% | 94288 | 0.089 | 0.08 | 8.4% | 15378 | 0.217 | 0.234 | 10.5% | 128395 | 0.173 | 0.142 |
| 2012 | 35-44 | M | 15.0% | 93926 | 0.089 | 0.082 | 13.0% | 9811 | 0.139 | 0.118 | 17.3% | 102879 | 0.138 | 0.118 |
| 2012 | 45-54 | F | 6.1% | 90484 | 0.086 | 0.073 | 7.2% | 8120 | 0.115 | 0.107 | 8.8% | 64339 | 0.086 | 0.055 |
| 2012 | 45-54 | M | 12.7% | 85779 | 0.081 | 0.062 | 10.1% | 5993 | 0.085 | 0.062 | 14.7% | 55061 | 0.074 | 0.05 |
| 2012 | 55-64 | F | 5.1% | 73200 | 0.069 | 0.052 | 5.5% | 2998 | 0.042 | 0.033 | 7.5% | 23085 | 0.031 | 0.014 |
| 2012 | 55-64 | M | 9.4% | 68448 | 0.065 | 0.041 | 8.0% | 2403 | 0.034 | 0.021 | 11.7% | 21335 | 0.029 | 0.015 |
| 2012 | >65 | F | 4.8% | 67236 | 0.064 | 0.052 | 4.4% | 685 | 0.01 | 0.005 | 7.4% | 5580 | 0.008 | 0.002 |
| 2012 | >65 | M | 6.6% | 52278 | 0.05 | 0.032 | 5.4% | 595 | 0.008 | 0.004 | 9.8% | 5104 | 0.007 | 0.003 |

*Numerator (number in subpopulation who received a test) and denominator (total number in population in the cohort) taken from NHLS data

** Numerator (number estimated in subpopulation) and denominator (total estimated population) taken from Thembisa 2012 estimates

Table A5 Test positivity of diagnostic TB tests stratified by HIV and viral suppression status, sex, and age for year 2013.

|  |  |  | People not living with HIV | | | | PLHIV virally suppressed | | | | PLHIV not virally suppressed | | | |
| --- | --- | --- | --- | --- | --- | --- | --- | --- | --- | --- | --- | --- | --- | --- |
| **Year** | **Age (years)** | **Sex** | **Test positivity** | **Number tested** | **Proportion of number tested*** | **Proportion of population**** | **Test positivity** | **Number tested** | **Proportion of number tested*** | **Proportion of population**** | **Test positivity** | **Number tested** | **Proportion of number tested*** | **Proportion of population**** |
| 2013 | 15-24 | F | 8.3% | 110229 | 0.104 | 0.139 | 9.2% | 4730 | 0.041 | 0.039 | 9.9% | 59244 | 0.084 | 0.13 |
| 2013 | 15-24 | M | 10.8% | 89215 | 0.084 | 0.15 | 9.8% | 1058 | 0.009 | 0.011 | 14.0% | 13722 | 0.019 | 0.026 |
| 2013 | 25-34 | F | 8.6% | 112930 | 0.106 | 0.104 | 8.8% | 24523 | 0.214 | 0.275 | 10.9% | 160186 | 0.227 | 0.282 |
| 2013 | 25-34 | M | 13.6% | 112844 | 0.106 | 0.132 | 12.5% | 8344 | 0.073 | 0.079 | 17.6% | 83151 | 0.118 | 0.151 |
| 2013 | 35-44 | F | 7.3% | 88992 | 0.084 | 0.078 | 6.8% | 24016 | 0.21 | 0.236 | 9.9% | 117884 | 0.167 | 0.141 |
| 2013 | 35-44 | M | 14.0% | 91035 | 0.086 | 0.082 | 11.4% | 15111 | 0.132 | 0.119 | 16.9% | 99144 | 0.14 | 0.122 |
| 2013 | 45-54 | F | 5.3% | 92036 | 0.087 | 0.072 | 5.6% | 13973 | 0.122 | 0.11 | 8.1% | 62475 | 0.088 | 0.058 |
| 2013 | 45-54 | M | 11.7% | 84585 | 0.08 | 0.062 | 8.5% | 10176 | 0.089 | 0.063 | 13.7% | 54821 | 0.078 | 0.053 |
| 2013 | 55-64 | F | 4.4% | 79082 | 0.074 | 0.053 | 4.7% | 5615 | 0.049 | 0.036 | 6.9% | 23379 | 0.033 | 0.016 |
| 2013 | 55-64 | M | 8.7% | 71684 | 0.067 | 0.042 | 6.7% | 4364 | 0.038 | 0.022 | 10.8% | 21730 | 0.031 | 0.017 |
| 2013 | >65 | F | 4.5% | 73235 | 0.069 | 0.053 | 4.1% | 1426 | 0.012 | 0.006 | 6.6% | 5794 | 0.008 | 0.002 |
| 2013 | >65 | M | 6.3% | 56373 | 0.053 | 0.033 | 5.4% | 1022 | 0.009 | 0.004 | 8.4% | 5512 | 0.008 | 0.003 |

*Numerator (number in subpopulation who received a test) and denominator (total number in population in the cohort) taken from NHLS data

** Numerator (number estimated in subpopulation) and denominator (total estimated population) taken from Thembisa 2012 estimates

Table A6 Test positivity of diagnostic TB tests stratified by HIV and viral suppression status, sex, and age for year 2014.

|  |  |  | People not living with HIV | | | | PLHIV virally suppressed | | | | PLHIV not virally suppressed | | | |
| --- | --- | --- | --- | --- | --- | --- | --- | --- | --- | --- | --- | --- | --- | --- |
| **Year** | **Age (years)** | **Sex** | **Test positivity** | **Number tested** | **Proportion of number tested*** | **Proportion of population**** | **Test positivity** | **Number tested** | **Proportion of number tested*** | **Proportion of population**** | **Test positivity** | **Number tested** | **Proportion of number tested*** | **Proportion of population**** |
| 2014 | 15-24 | F | 7.8% | 108096 | 0.101 | 0.137 | 8.9% | 6618 | 0.042 | 0.038 | 9.7% | 56754 | 0.081 | 0.123 |
| 2014 | 15-24 | M | 10.1% | 95250 | 0.089 | 0.147 | 9.1% | 1567 | 0.01 | 0.012 | 13.3% | 15363 | 0.022 | 0.025 |
| 2014 | 25-34 | F | 8.0% | 105724 | 0.099 | 0.106 | 8.2% | 32321 | 0.207 | 0.26 | 11.1% | 148356 | 0.213 | 0.276 |
| 2014 | 25-34 | M | 12.8% | 118254 | 0.111 | 0.135 | 12.0% | 11307 | 0.072 | 0.074 | 17.4% | 85162 | 0.122 | 0.151 |
| 2014 | 35-44 | F | 6.8% | 82813 | 0.078 | 0.076 | 6.7% | 32131 | 0.205 | 0.24 | 10.1% | 112736 | 0.162 | 0.141 |
| 2014 | 35-44 | M | 13.2% | 90309 | 0.085 | 0.083 | 10.6% | 20984 | 0.134 | 0.121 | 17.0% | 101267 | 0.145 | 0.127 |
| 2014 | 45-54 | F | 4.9% | 90084 | 0.084 | 0.071 | 5.3% | 19130 | 0.122 | 0.115 | 8.2% | 62009 | 0.089 | 0.059 |
| 2014 | 45-54 | M | 11.4% | 84025 | 0.079 | 0.062 | 8.6% | 14251 | 0.091 | 0.065 | 14.4% | 56766 | 0.081 | 0.056 |
| 2014 | 55-64 | F | 4.2% | 80621 | 0.076 | 0.054 | 4.2% | 8105 | 0.052 | 0.04 | 6.9% | 23784 | 0.034 | 0.017 |
| 2014 | 55-64 | M | 8.5% | 73556 | 0.069 | 0.042 | 6.4% | 6340 | 0.041 | 0.023 | 10.9% | 22424 | 0.032 | 0.019 |
| 2014 | >65 | F | 4.3% | 77665 | 0.073 | 0.054 | 3.5% | 2075 | 0.013 | 0.007 | 6.6% | 6158 | 0.009 | 0.003 |
| 2014 | >65 | M | 6.1% | 60795 | 0.057 | 0.034 | 5.1% | 1684 | 0.011 | 0.005 | 8.4% | 5821 | 0.008 | 0.004 |

*Numerator (number in subpopulation who received a test) and denominator (total number in population in the cohort) taken from NHLS data

** Numerator (number estimated in subpopulation) and denominator (total estimated population) taken from Thembisa 2012 estimates

Table A7 Test positivity of diagnostic TB tests stratified by HIV and viral suppression status, sex, and age for year 2015.

|  |  |  | People not living with HIV | | | | PLHIV virally suppressed | | | | PLHIV not virally suppressed | | | |
| --- | --- | --- | --- | --- | --- | --- | --- | --- | --- | --- | --- | --- | --- | --- |
| **Year** | **Age (years)** | **Sex** | **Test positivity** | **Number tested** | **Proportion of number tested*** | **Proportion of population**** | **Test positivity** | **Number tested** | **Proportion of number tested*** | **Proportion of population**** | **Test positivity** | **Number tested** | **Proportion of number tested*** | **Proportion of population**** |
| 2015 | 15-24 | F | 7.5% | 106796 | 0.094 | 0.134 | 8.5% | 9369 | 0.046 | 0.041 | 8.9% | 57125 | 0.082 | 0.114 |
| 2015 | 15-24 | M | 9.5% | 100042 | 0.088 | 0.144 | 9.3% | 2218 | 0.011 | 0.013 | 12.0% | 17159 | 0.024 | 0.026 |
| 2015 | 25-34 | F | 7.4% | 106575 | 0.093 | 0.108 | 7.9% | 41355 | 0.205 | 0.249 | 10.2% | 142921 | 0.204 | 0.263 |
| 2015 | 25-34 | M | 11.0% | 135765 | 0.119 | 0.137 | 11.8% | 14391 | 0.071 | 0.071 | 16.1% | 89039 | 0.127 | 0.15 |
| 2015 | 35-44 | F | 6.3% | 82585 | 0.072 | 0.075 | 6.4% | 40156 | 0.199 | 0.241 | 9.6% | 109462 | 0.156 | 0.142 |
| 2015 | 35-44 | M | 11.8% | 99207 | 0.087 | 0.084 | 10.4% | 26626 | 0.132 | 0.12 | 16.1% | 102102 | 0.146 | 0.133 |
| 2015 | 45-54 | F | 4.5% | 94365 | 0.083 | 0.07 | 4.9% | 24261 | 0.12 | 0.118 | 7.6% | 62668 | 0.089 | 0.062 |
| 2015 | 45-54 | M | 10.7% | 89697 | 0.079 | 0.061 | 8.5% | 18704 | 0.093 | 0.066 | 13.6% | 57726 | 0.082 | 0.06 |
| 2015 | 55-64 | F | 3.7% | 88540 | 0.078 | 0.054 | 4.3% | 10552 | 0.052 | 0.042 | 6.6% | 25311 | 0.036 | 0.02 |
| 2015 | 55-64 | M | 8.2% | 80866 | 0.071 | 0.043 | 6.5% | 8638 | 0.043 | 0.025 | 10.7% | 23853 | 0.034 | 0.021 |
| 2015 | >65 | F | 4.0% | 88042 | 0.077 | 0.055 | 3.1% | 2981 | 0.015 | 0.008 | 6.1% | 7100 | 0.01 | 0.003 |
| 2015 | >65 | M | 5.8% | 67573 | 0.059 | 0.034 | 4.5% | 2423 | 0.012 | 0.005 | 8.4% | 6372 | 0.009 | 0.004 |

*Numerator (number in subpopulation who received a test) and denominator (total number in population in the cohort) taken from NHLS data

** Numerator (number estimated in subpopulation) and denominator (total estimated population) taken from Thembisa 2012 estimates

Table A8 Test positivity of diagnostic TB tests stratified by HIV and viral suppression status, sex, and age for year 2016.

|  |  |  | People not living with HIV | | | | PLHIV virally suppressed | | | | PLHIV not virally suppressed | | | |
| --- | --- | --- | --- | --- | --- | --- | --- | --- | --- | --- | --- | --- | --- | --- |
| **Year** | **Age (years)** | **Sex** | **Test positivity** | **Number tested** | **Proportion of number tested*** | **Proportion of population**** | **Test positivity** | **Number tested** | **Proportion of number tested*** | **Proportion of population**** | **Test positivity** | **Number tested** | **Proportion of number tested*** | **Proportion of population**** |
| 2016 | 15-24 | F | 7.7% | 92459 | 0.091 | 0.131 | 7.8% | 10012 | 0.047 | 0.044 | 8.5% | 50981 | 0.08 | 0.107 |
| 2016 | 15-24 | M | 10.0% | 89326 | 0.088 | 0.141 | 8.8% | 2397 | 0.011 | 0.014 | 11.7% | 16288 | 0.026 | 0.027 |
| 2016 | 25-34 | F | 7.6% | 92295 | 0.091 | 0.109 | 7.8% | 43507 | 0.205 | 0.239 | 10.4% | 124513 | 0.196 | 0.247 |
| 2016 | 25-34 | M | 11.8% | 120561 | 0.119 | 0.138 | 11.3% | 14999 | 0.071 | 0.069 | 16.0% | 84297 | 0.133 | 0.147 |
| 2016 | 35-44 | F | 6.2% | 69864 | 0.069 | 0.075 | 6.4% | 41184 | 0.194 | 0.242 | 9.7% | 94199 | 0.149 | 0.145 |
| 2016 | 35-44 | M | 12.3% | 87626 | 0.087 | 0.086 | 10.7% | 27665 | 0.13 | 0.12 | 16.0% | 95806 | 0.151 | 0.14 |
| 2016 | 45-54 | F | 4.7% | 80131 | 0.079 | 0.069 | 5.2% | 25151 | 0.119 | 0.12 | 7.6% | 55401 | 0.087 | 0.067 |
| 2016 | 45-54 | M | 10.8% | 79001 | 0.078 | 0.061 | 8.5% | 20192 | 0.095 | 0.068 | 13.3% | 54086 | 0.085 | 0.065 |
| 2016 | 55-64 | F | 3.6% | 79287 | 0.078 | 0.055 | 3.7% | 11354 | 0.054 | 0.045 | 6.5% | 22897 | 0.036 | 0.023 |
| 2016 | 55-64 | M | 8.2% | 75499 | 0.075 | 0.044 | 6.3% | 9633 | 0.045 | 0.026 | 10.3% | 22460 | 0.035 | 0.024 |
| 2016 | >65 | F | 4.1% | 82245 | 0.081 | 0.056 | 3.3% | 3303 | 0.016 | 0.009 | 5.6% | 6692 | 0.011 | 0.004 |
| 2016 | >65 | M | 5.6% | 64571 | 0.064 | 0.035 | 5.1% | 2784 | 0.013 | 0.006 | 7.6% | 6534 | 0.01 | 0.005 |

*Numerator (number in subpopulation who received a test) and denominator (total number in population in the cohort) taken from NHLS data

** Numerator (number estimated in subpopulation) and denominator (total estimated population) taken from Thembisa 2012 estimates

Table A9 Test positivity of diagnostic TB tests stratified by HIV and viral suppression status and province.

|  | People not living with HIV | | | | PLHIV virally suppressed | | | | PLHIV not virally suppressed | | | |
| --- | --- | --- | --- | --- | --- | --- | --- | --- | --- | --- | --- | --- |
| **Province** | **Test positivity** | **Number tested** | **Proportion of number tested*** | **Proportion of population**** | **Test positivity** | **Number tested** | **Proportion of number tested*** | **Proportion of population**** | **Test positivity** | **Number tested** | **Proportion of number tested*** | **Proportion of population**** |
| EC | 10.31% | 977130 | 0.684 | 0.881 | 11.85% | 95064 | 0.062 | 0.048 | 16.39% | 392837 | 0.254 | 0.070 |
| FS | 8.72% | 243313 | 0.525 | 0.852 | 9.45% | 44917 | 0.090 | 0.060 | 12.53% | 192033 | 0.385 | 0.089 |
| GP | 10.18% | 664100 | 0.48 | 0.888 | 10.21% | 104219 | 0.072 | 0.046 | 12.54% | 651687 | 0.449 | 0.065 |
| KZN | 9.17% | 1147547 | 0.546 | 0.808 | 8.80% | 216844 | 0.096 | 0.088 | 10.99% | 811969 | 0.358 | 0.104 |
| LP | 5.41% | 517080 | 0.664 | 0.897 | 7.48% | 53050 | 0.064 | 0.040 | 10.76% | 226460 | 0.272 | 0.064 |
| MP | 10.48% | 213260 | 0.473 | 0.843 | 12.16% | 45309 | 0.095 | 0.062 | 14.35% | 206898 | 0.433 | 0.095 |
| NC | 10.78% | 147348 | 0.685 | 0.912 | 11.30% | 13291 | 0.057 | 0.033 | 15.58% | 59966 | 0.258 | 0.055 |
| NW | 8.56% | 292649 | 0.537 | 0.864 | 9.74% | 44928 | 0.077 | 0.050 | 12.34% | 224942 | 0.386 | 0.086 |
| WC | 15.52% | 543557 | 0.707 | 0.937 | 16.40% | 49076 | 0.059 | 0.026 | 21.43% | 195966 | 0.234 | 0.037 |

*Numerator (number in subpopulation who received a test) and denominator (total number in population in the cohort) taken from NHLS data

** Numerator (number estimated in subpopulation) and denominator (total estimated population) taken from Thembisa 2012 estimates

Table A10 Test positivity of diagnostic TB tests stratified by HIV and viral suppression status, sex, and age for Eastern Cape.

|  |  |  | People not living with HIV | | | | PLHIV virally suppressed | | | | PLHIV not virally suppressed | | | |
| --- | --- | --- | --- | --- | --- | --- | --- | --- | --- | --- | --- | --- | --- | --- |
| **Province** | **Age (years)** | **Sex** | **Test positivity** | **Number tested** | **Proportion of number tested*** | **Proportion of population**** | **Test positivity** | **Number tested** | **Proportion of number tested*** | **Proportion of population**** | **Test positivity** | **Number tested** | **Proportion of number tested*** | **Proportion of population**** |
| EC | 15-24 | F | 8.9% | 101470 | 0.104 | 0.155 | 14.8% | 5108 | 0.054 | 0.037 | 14.1% | 39324 | 0.1 | 0.144 |
| EC | 15-24 | M | 12.9% | 91134 | 0.093 | 0.166 | 16.7% | 1021 | 0.011 | 0.01 | 19.3% | 9353 | 0.024 | 0.025 |
| EC | 25-34 | F | 8.6% | 71852 | 0.074 | 0.085 | 12.4% | 20831 | 0.219 | 0.285 | 15.1% | 85505 | 0.218 | 0.306 |
| EC | 25-34 | M | 17.4% | 86511 | 0.089 | 0.102 | 17.7% | 6529 | 0.069 | 0.077 | 23.3% | 43849 | 0.112 | 0.119 |
| EC | 35-44 | F | 7.6% | 61193 | 0.063 | 0.071 | 10.0% | 18658 | 0.196 | 0.249 | 13.3% | 62406 | 0.159 | 0.164 |
| EC | 35-44 | M | 17.4% | 67324 | 0.069 | 0.064 | 16.7% | 11200 | 0.118 | 0.099 | 22.0% | 49983 | 0.127 | 0.086 |
| EC | 45-54 | F | 5.9% | 80832 | 0.083 | 0.079 | 7.9% | 11236 | 0.118 | 0.123 | 11.1% | 35578 | 0.091 | 0.074 |
| EC | 45-54 | M | 14.9% | 71322 | 0.073 | 0.056 | 12.5% | 7890 | 0.083 | 0.055 | 18.6% | 29000 | 0.074 | 0.043 |
| EC | 55-64 | F | 5.1% | 93811 | 0.096 | 0.064 | 5.5% | 5335 | 0.056 | 0.037 | 9.8% | 15336 | 0.039 | 0.019 |
| EC | 55-64 | M | 10.8% | 75563 | 0.077 | 0.045 | 9.3% | 4362 | 0.046 | 0.02 | 14.0% | 14011 | 0.036 | 0.015 |
| EC | >65 | F | 6.3% | 101941 | 0.104 | 0.071 | 5.7% | 1539 | 0.016 | 0.005 | 10.0% | 4337 | 0.011 | 0.002 |
| EC | >65 | M | 8.0% | 74177 | 0.076 | 0.04 | 6.7% | 1355 | 0.014 | 0.003 | 11.7% | 4155 | 0.011 | 0.002 |

*Numerator (number in subpopulation who received a test) and denominator (total number in population in the cohort) taken from NHLS data

** Numerator (number estimated in subpopulation) and denominator (total estimated population) taken from Thembisa 2012 estimates

Table A11 Test positivity of diagnostic TB tests stratified by HIV and viral suppression status, sex, and age for Free State.

|  |  |  | People not living with HIV | | | | PLHIV virally suppressed | | | | PLHIV not virally suppressed | | | |
| --- | --- | --- | --- | --- | --- | --- | --- | --- | --- | --- | --- | --- | --- | --- |
| **Province** | **Age (years)** | **Sex** | **Test positivity** | **Number tested** | **Proportion of number tested*** | **Proportion of population**** | **Test positivity** | **Number tested** | **Proportion of number tested*** | **Proportion of population**** | **Test positivity** | **Number tested** | **Proportion of number tested*** | **Proportion of population**** |
| FS | 15-24 | F | 9.4% | 21544 | 0.089 | 0.145 | 11.6% | 1890 | 0.042 | 0.035 | 11.5% | 14127 | 0.074 | 0.124 |
| FS | 15-24 | M | 10.2% | 22527 | 0.093 | 0.159 | 11.1% | 498 | 0.011 | 0.01 | 13.7% | 4387 | 0.023 | 0.025 |
| FS | 25-34 | F | 8.4% | 19804 | 0.081 | 0.096 | 9.3% | 8058 | 0.179 | 0.26 | 11.5% | 35616 | 0.185 | 0.278 |
| FS | 25-34 | M | 12.6% | 28253 | 0.116 | 0.124 | 13.5% | 3575 | 0.08 | 0.08 | 17.0% | 23904 | 0.124 | 0.144 |
| FS | 35-44 | F | 6.8% | 16432 | 0.068 | 0.075 | 8.2% | 8554 | 0.19 | 0.234 | 10.2% | 30415 | 0.158 | 0.149 |
| FS | 35-44 | M | 13.2% | 21065 | 0.087 | 0.074 | 12.8% | 6300 | 0.14 | 0.114 | 16.3% | 28298 | 0.147 | 0.109 |
| FS | 45-54 | F | 4.9% | 20707 | 0.085 | 0.072 | 6.3% | 5656 | 0.126 | 0.123 | 8.1% | 19400 | 0.101 | 0.068 |
| FS | 45-54 | M | 11.6% | 21371 | 0.088 | 0.065 | 10.3% | 4774 | 0.106 | 0.067 | 14.1% | 17352 | 0.09 | 0.055 |
| FS | 55-64 | F | 3.9% | 19733 | 0.081 | 0.055 | 4.8% | 2504 | 0.056 | 0.042 | 7.1% | 7788 | 0.041 | 0.021 |
| FS | 55-64 | M | 8.7% | 19860 | 0.082 | 0.043 | 8.1% | 2069 | 0.046 | 0.023 | 11.7% | 7167 | 0.037 | 0.019 |
| FS | >65 | F | 4.4% | 17380 | 0.071 | 0.058 | 3.6% | 532 | 0.012 | 0.008 | 7.0% | 1777 | 0.009 | 0.003 |
| FS | >65 | M | 6.3% | 14637 | 0.06 | 0.035 | 5.0% | 507 | 0.011 | 0.005 | 8.3% | 1802 | 0.009 | 0.004 |

*Numerator (number in subpopulation who received a test) and denominator (total number in population in the cohort) taken from NHLS data

** Numerator (number estimated in subpopulation) and denominator (total estimated population) taken from Thembisa 2012 estimates

Table A12 Test positivity of diagnostic TB tests stratified by HIV and viral suppression status, sex, and age for Gauteng.

|  |  |  | People not living with HIV | | | | PLHIV virally suppressed | | | | PLHIV not virally suppressed | | | |
| --- | --- | --- | --- | --- | --- | --- | --- | --- | --- | --- | --- | --- | --- | --- |
| **Province** | **Age (years)** | **Sex** | **Test positivity** | **Number tested** | **Proportion of number tested*** | **Proportion of population**** | **Test positivity** | **Number tested** | **Proportion of number tested*** | **Proportion of population**** | **Test positivity** | **Number tested** | **Proportion of number tested*** | **Proportion of population**** |
| GP | 15-24 | F | 10.6% | 47845 | 0.072 | 0.121 | 11.2% | 3528 | 0.034 | 0.032 | 10.2% | 39653 | 0.061 | 0.115 |
| GP | 15-24 | M | 10.9% | 46886 | 0.071 | 0.13 | 11.6% | 1041 | 0.01 | 0.01 | 13.3% | 11485 | 0.018 | 0.02 |
| GP | 25-34 | F | 10.4% | 80270 | 0.121 | 0.119 | 10.1% | 20360 | 0.195 | 0.27 | 11.0% | 137425 | 0.211 | 0.295 |
| GP | 25-34 | M | 12.3% | 97986 | 0.148 | 0.156 | 13.1% | 7983 | 0.077 | 0.079 | 15.9% | 79845 | 0.123 | 0.143 |
| GP | 35-44 | F | 8.8% | 61422 | 0.092 | 0.086 | 8.7% | 21677 | 0.208 | 0.226 | 10.5% | 111900 | 0.172 | 0.145 |
| GP | 35-44 | M | 13.4% | 79766 | 0.12 | 0.102 | 12.3% | 16571 | 0.159 | 0.136 | 16.1% | 107582 | 0.165 | 0.133 |
| GP | 45-54 | F | 6.5% | 50620 | 0.076 | 0.069 | 7.8% | 11743 | 0.113 | 0.105 | 9.0% | 56710 | 0.087 | 0.057 |
| GP | 45-54 | M | 12.9% | 57682 | 0.087 | 0.068 | 12.1% | 10849 | 0.104 | 0.075 | 14.9% | 57847 | 0.089 | 0.056 |
| GP | 55-64 | F | 5.4% | 38929 | 0.059 | 0.045 | 6.0% | 4481 | 0.043 | 0.034 | 7.8% | 19828 | 0.03 | 0.015 |
| GP | 55-64 | M | 10.3% | 44718 | 0.067 | 0.041 | 9.4% | 4276 | 0.041 | 0.025 | 12.0% | 20815 | 0.032 | 0.016 |
| GP | >65 | F | 4.5% | 29716 | 0.045 | 0.038 | 4.3% | 852 | 0.008 | 0.005 | 7.2% | 4129 | 0.006 | 0.002 |
| GP | >65 | M | 6.7% | 28260 | 0.043 | 0.026 | 8.8% | 858 | 0.008 | 0.004 | 9.8% | 4468 | 0.007 | 0.003 |

*Numerator (number in subpopulation who received a test) and denominator (total number in population in the cohort) taken from NHLS data

** Numerator (number estimated in subpopulation) and denominator (total estimated population) taken from Thembisa 2012 estimates

Table A13 Test positivity of diagnostic TB tests stratified by HIV and viral suppression status, sex, and age for KwaZulu-Natal.

|  |  |  | People not living with HIV | | | | PLHIV virally suppressed | | | | PLHIV not virally suppressed | | | |
| --- | --- | --- | --- | --- | --- | --- | --- | --- | --- | --- | --- | --- | --- | --- |
| **Province** | **Age (years)** | **Sex** | **Test positivity** | **Number tested** | **Proportion of number tested*** | **Proportion of population**** | **Test positivity** | **Number tested** | **Proportion of number tested*** | **Proportion of population**** | **Test positivity** | **Number tested** | **Proportion of number tested*** | **Proportion of population**** |
| KZN | 15-24 | F | 8.2% | 141350 | 0.123 | 0.169 | 9.4% | 13343 | 0.062 | 0.045 | 8.2% | 94008 | 0.116 | 0.157 |
| KZN | 15-24 | M | 11.7% | 97730 | 0.085 | 0.184 | 10.9% | 3181 | 0.015 | 0.012 | 13.0% | 22966 | 0.028 | 0.034 |
| KZN | 25-34 | F | 8.6% | 160038 | 0.139 | 0.092 | 8.4% | 50719 | 0.234 | 0.288 | 9.2% | 200794 | 0.247 | 0.276 |
| KZN | 25-34 | M | 14.0% | 135989 | 0.119 | 0.124 | 12.2% | 19991 | 0.092 | 0.091 | 15.3% | 119835 | 0.148 | 0.167 |
| KZN | 35-44 | F | 7.0% | 106765 | 0.093 | 0.069 | 7.1% | 42225 | 0.195 | 0.232 | 8.5% | 117741 | 0.145 | 0.127 |
| KZN | 35-44 | M | 13.8% | 97876 | 0.085 | 0.065 | 11.6% | 29546 | 0.136 | 0.112 | 15.1% | 106730 | 0.131 | 0.106 |
| KZN | 45-54 | F | 4.8% | 91875 | 0.08 | 0.07 | 5.7% | 22420 | 0.103 | 0.109 | 7.0% | 56557 | 0.07 | 0.054 |
| KZN | 45-54 | M | 12.2% | 70101 | 0.061 | 0.049 | 9.6% | 16418 | 0.076 | 0.054 | 13.3% | 46625 | 0.057 | 0.045 |
| KZN | 55-64 | F | 4.1% | 75851 | 0.066 | 0.055 | 4.9% | 8494 | 0.039 | 0.032 | 6.1% | 20025 | 0.025 | 0.014 |
| KZN | 55-64 | M | 9.5% | 56966 | 0.05 | 0.036 | 8.1% | 6541 | 0.03 | 0.018 | 11.0% | 17121 | 0.021 | 0.015 |
| KZN | >65 | F | 4.1% | 70063 | 0.061 | 0.055 | 4.1% | 2182 | 0.01 | 0.004 | 6.1% | 5153 | 0.006 | 0.002 |
| KZN | >65 | M | 6.5% | 42943 | 0.037 | 0.032 | 5.9% | 1784 | 0.008 | 0.003 | 8.9% | 4414 | 0.005 | 0.003 |

*Numerator (number in subpopulation who received a test) and denominator (total number in population in the cohort) taken from NHLS data

** Numerator (number estimated in subpopulation) and denominator (total estimated population) taken from Thembisa 2012 estimates

Table A14 Test positivity of diagnostic TB tests stratified by HIV and viral suppression status, sex, and age for Limpopo.

|  |  |  | People not living with HIV | | | | PLHIV virally suppressed | | | | PLHIV not virally suppressed | | | |
| --- | --- | --- | --- | --- | --- | --- | --- | --- | --- | --- | --- | --- | --- | --- |
| **Province** | **Age (years)** | **Sex** | **Test positivity** | **Number tested** | **Proportion of number tested*** | **Proportion of population**** | **Test positivity** | **Number tested** | **Proportion of number tested*** | **Proportion of population**** | **Test positivity** | **Number tested** | **Proportion of number tested*** | **Proportion of population**** |
| LP | 15-24 | F | 4.4% | 70589 | 0.137 | 0.165 | 8.1% | 1988 | 0.037 | 0.026 | 9.4% | 16211 | 0.072 | 0.094 |
| LP | 15-24 | M | 5.2% | 53168 | 0.103 | 0.174 | 6.2% | 418 | 0.008 | 0.008 | 10.6% | 3484 | 0.015 | 0.017 |
| LP | 25-34 | F | 4.7% | 55704 | 0.108 | 0.104 | 7.8% | 10626 | 0.2 | 0.256 | 10.2% | 47257 | 0.209 | 0.311 |
| LP | 25-34 | M | 8.9% | 43959 | 0.085 | 0.113 | 11.1% | 1931 | 0.036 | 0.057 | 16.1% | 16176 | 0.071 | 0.102 |
| LP | 35-44 | F | 4.2% | 41715 | 0.081 | 0.073 | 6.5% | 12097 | 0.228 | 0.297 | 9.3% | 43041 | 0.19 | 0.21 |
| LP | 35-44 | M | 11.1% | 32458 | 0.063 | 0.063 | 12.4% | 4976 | 0.094 | 0.101 | 16.2% | 27438 | 0.121 | 0.098 |
| LP | 45-54 | F | 2.9% | 42512 | 0.082 | 0.068 | 5.3% | 7727 | 0.146 | 0.135 | 7.0% | 25407 | 0.112 | 0.081 |
| LP | 45-54 | M | 10.1% | 29703 | 0.057 | 0.049 | 10.0% | 4433 | 0.084 | 0.056 | 13.4% | 18940 | 0.084 | 0.047 |
| LP | 55-64 | F | 2.5% | 38129 | 0.074 | 0.051 | 3.9% | 3820 | 0.072 | 0.037 | 5.4% | 11324 | 0.05 | 0.019 |
| LP | 55-64 | M | 7.7% | 28802 | 0.056 | 0.035 | 7.3% | 2487 | 0.047 | 0.018 | 10.2% | 9220 | 0.041 | 0.014 |
| LP | >65 | F | 2.2% | 48952 | 0.095 | 0.072 | 2.6% | 1649 | 0.031 | 0.007 | 4.6% | 4700 | 0.021 | 0.003 |
| LP | >65 | M | 4.8% | 31389 | 0.061 | 0.034 | 4.8% | 898 | 0.017 | 0.003 | 7.3% | 3262 | 0.014 | 0.003 |

*Numerator (number in subpopulation who received a test) and denominator (total number in population in the cohort) taken from NHLS data

** Numerator (number estimated in subpopulation) and denominator (total estimated population) taken from Thembisa 2012 estimates

Table A15 Test positivity of diagnostic TB tests stratified by HIV and viral suppression status, sex, and age for Mpumalanga.

|  |  |  | People not living with HIV | | | | PLHIV virally suppressed | | | | PLHIV not virally suppressed | | | |
| --- | --- | --- | --- | --- | --- | --- | --- | --- | --- | --- | --- | --- | --- | --- |
| **Province** | **Age (years)** | **Sex** | **Test positivity** | **Number tested** | **Proportion of number tested*** | **Proportion of population**** | **Test positivity** | **Number tested** | **Proportion of number tested*** | **Proportion of population**** | **Test positivity** | **Number tested** | **Proportion of number tested*** | **Proportion of population**** |
| MP | 15-24 | F | 11.3% | 19101 | 0.09 | 0.152 | 14.0% | 1843 | 0.041 | 0.044 | 13.6% | 15683 | 0.076 | 0.152 |
| MP | 15-24 | M | 11.8% | 19032 | 0.089 | 0.172 | 14.4% | 482 | 0.011 | 0.012 | 15.4% | 4572 | 0.022 | 0.031 |
| MP | 25-34 | F | 10.7% | 21024 | 0.099 | 0.095 | 13.5% | 8968 | 0.198 | 0.287 | 13.7% | 41970 | 0.203 | 0.29 |
| MP | 25-34 | M | 14.0% | 27423 | 0.129 | 0.129 | 16.1% | 3508 | 0.077 | 0.081 | 18.9% | 24895 | 0.12 | 0.145 |
| MP | 35-44 | F | 8.8% | 16465 | 0.077 | 0.075 | 10.1% | 8660 | 0.191 | 0.239 | 11.9% | 32104 | 0.155 | 0.14 |
| MP | 35-44 | M | 15.4% | 19806 | 0.093 | 0.078 | 15.6% | 6066 | 0.134 | 0.112 | 18.8% | 30571 | 0.148 | 0.107 |
| MP | 45-54 | F | 5.9% | 16823 | 0.079 | 0.069 | 8.7% | 5449 | 0.12 | 0.107 | 8.7% | 18533 | 0.09 | 0.055 |
| MP | 45-54 | M | 14.1% | 16816 | 0.079 | 0.061 | 13.6% | 4291 | 0.095 | 0.06 | 16.3% | 17705 | 0.086 | 0.048 |
| MP | 55-64 | F | 5.0% | 15003 | 0.07 | 0.05 | 5.2% | 2542 | 0.056 | 0.032 | 7.6% | 8182 | 0.04 | 0.014 |
| MP | 55-64 | M | 10.8% | 15003 | 0.07 | 0.041 | 11.1% | 2038 | 0.045 | 0.019 | 12.8% | 7800 | 0.038 | 0.014 |
| MP | >65 | F | 4.4% | 14356 | 0.067 | 0.049 | 5.0% | 817 | 0.018 | 0.004 | 6.5% | 2459 | 0.012 | 0.002 |
| MP | >65 | M | 7.1% | 12408 | 0.058 | 0.029 | 5.8% | 645 | 0.014 | 0.003 | 9.2% | 2424 | 0.012 | 0.002 |

*Numerator (number in subpopulation who received a test) and denominator (total number in population in the cohort) taken from NHLS data

** Numerator (number estimated in subpopulation) and denominator (total estimated population) taken from Thembisa 2012 estimates

Table A16 Test positivity of diagnostic TB tests stratified by HIV and viral suppression status, sex, and age for Northern Cape.

|  |  |  | People not living with HIV | | | | PLHIV virally suppressed | | | | PLHIV not virally suppressed | | | |
| --- | --- | --- | --- | --- | --- | --- | --- | --- | --- | --- | --- | --- | --- | --- |
| **Province** | **Age (years)** | **Sex** | **Test positivity** | **Number tested** | **Proportion of number tested*** | **Proportion of population**** | **Test positivity** | **Number tested** | **Proportion of number tested*** | **Proportion of population**** | **Test positivity** | **Number tested** | **Proportion of number tested*** | **Proportion of population**** |
| NC | 15-24 | F | 10.5% | 14134 | 0.096 | 0.139 | 11.9% | 556 | 0.042 | 0.031 | 14.7% | 4181 | 0.07 | 0.108 |
| NC | 15-24 | M | 12.3% | 14064 | 0.095 | 0.153 | 12.3% | 165 | 0.012 | 0.008 | 16.4% | 1457 | 0.024 | 0.021 |
| NC | 25-34 | F | 9.5% | 12375 | 0.084 | 0.099 | 12.5% | 2554 | 0.192 | 0.271 | 16.1% | 10896 | 0.182 | 0.289 |
| NC | 25-34 | M | 14.9% | 16770 | 0.114 | 0.121 | 15.7% | 1185 | 0.089 | 0.088 | 20.8% | 7618 | 0.127 | 0.145 |
| NC | 35-44 | F | 8.9% | 11813 | 0.08 | 0.076 | 10.4% | 2580 | 0.194 | 0.245 | 13.7% | 9526 | 0.159 | 0.165 |
| NC | 35-44 | M | 15.5% | 14468 | 0.098 | 0.079 | 13.8% | 1961 | 0.148 | 0.119 | 18.4% | 9447 | 0.158 | 0.111 |
| NC | 45-54 | F | 7.0% | 13487 | 0.092 | 0.072 | 7.3% | 1478 | 0.111 | 0.114 | 10.6% | 5568 | 0.093 | 0.068 |
| NC | 45-54 | M | 13.4% | 14522 | 0.099 | 0.065 | 11.0% | 1428 | 0.107 | 0.061 | 14.7% | 5835 | 0.097 | 0.05 |
| NC | 55-64 | F | 5.5% | 10321 | 0.07 | 0.053 | 6.7% | 603 | 0.045 | 0.034 | 9.4% | 2101 | 0.035 | 0.02 |
| NC | 55-64 | M | 11.1% | 10847 | 0.074 | 0.044 | 8.4% | 521 | 0.039 | 0.02 | 13.2% | 2185 | 0.036 | 0.016 |
| NC | >65 | F | 5.0% | 7446 | 0.051 | 0.06 | 0.5% | 124 | 0.009 | 0.006 | 9.3% | 512 | 0.009 | 0.003 |
| NC | >65 | M | 7.8% | 7101 | 0.048 | 0.039 | 6.1% | 136 | 0.01 | 0.004 | 8.8% | 640 | 0.011 | 0.004 |

*Numerator (number in subpopulation who received a test) and denominator (total number in population in the cohort) taken from NHLS data

** Numerator (number estimated in subpopulation) and denominator (total estimated population) taken from Thembisa 2012 estimates

Table A17 Test positivity of diagnostic TB tests stratified by HIV and viral suppression status, sex, and age for North West.

|  |  |  | People not living with HIV | | | | PLHIV virally suppressed | | | | PLHIV not virally suppressed | | | |
| --- | --- | --- | --- | --- | --- | --- | --- | --- | --- | --- | --- | --- | --- | --- |
| **Province** | **Age (years)** | **Sex** | **Test positivity** | **Number tested** | **Proportion of number tested*** | **Proportion of population**** | **Test positivity** | **Number tested** | **Proportion of number tested*** | **Proportion of population**** | **Test positivity** | **Number tested** | **Proportion of number tested*** | **Proportion of population**** |
| NW | 15-24 | F | 8.3% | 28468 | 0.097 | 0.136 | 10.1% | 1982 | 0.044 | 0.029 | 10.2% | 15744 | 0.07 | 0.109 |
| NW | 15-24 | M | 9.5% | 26263 | 0.09 | 0.157 | 13.0% | 449 | 0.01 | 0.008 | 13.6% | 4374 | 0.019 | 0.022 |
| NW | 25-34 | F | 7.4% | 26848 | 0.092 | 0.093 | 9.1% | 8815 | 0.196 | 0.257 | 11.1% | 43927 | 0.195 | 0.27 |
| NW | 25-34 | M | 11.8% | 32318 | 0.11 | 0.134 | 15.2% | 2963 | 0.066 | 0.072 | 16.9% | 24137 | 0.107 | 0.147 |
| NW | 35-44 | F | 6.7% | 19804 | 0.068 | 0.071 | 8.2% | 8717 | 0.194 | 0.247 | 10.5% | 37127 | 0.165 | 0.154 |
| NW | 35-44 | M | 13.4% | 25757 | 0.088 | 0.087 | 13.9% | 5945 | 0.132 | 0.123 | 16.5% | 33524 | 0.149 | 0.13 |
| NW | 45-54 | F | 5.2% | 22078 | 0.075 | 0.064 | 6.8% | 5380 | 0.12 | 0.122 | 8.3% | 21748 | 0.097 | 0.064 |
| NW | 45-54 | M | 11.9% | 26108 | 0.089 | 0.071 | 11.1% | 4622 | 0.103 | 0.071 | 14.1% | 21420 | 0.095 | 0.062 |
| NW | 55-64 | F | 4.3% | 20549 | 0.07 | 0.049 | 5.2% | 2582 | 0.057 | 0.038 | 7.1% | 9146 | 0.041 | 0.017 |
| NW | 55-64 | M | 9.5% | 24786 | 0.085 | 0.046 | 8.0% | 2335 | 0.052 | 0.023 | 11.2% | 9198 | 0.041 | 0.019 |
| NW | >65 | F | 3.8% | 19437 | 0.066 | 0.054 | 3.8% | 601 | 0.013 | 0.006 | 5.9% | 2323 | 0.01 | 0.002 |
| NW | >65 | M | 6.1% | 20233 | 0.069 | 0.037 | 5.2% | 537 | 0.012 | 0.004 | 8.4% | 2274 | 0.01 | 0.003 |

*Numerator (number in subpopulation who received a test) and denominator (total number in population in the cohort) taken from NHLS data

** Numerator (number estimated in subpopulation) and denominator (total estimated population) taken from Thembisa 2012 estimates

Table A18 Test positivity of diagnostic TB tests stratified by HIV and viral suppression status, sex, and age for Western Cape.

|  |  |  | People not living with HIV | | | | PLHIV virally suppressed | | | | PLHIV not virally suppressed | | | |
| --- | --- | --- | --- | --- | --- | --- | --- | --- | --- | --- | --- | --- | --- | --- |
| **Province** | **Age (years)** | **Sex** | **Test positivity** | **Number tested** | **Proportion of number tested*** | **Proportion of population**** | **Test positivity** | **Number tested** | **Proportion of number tested*** | **Proportion of population**** | **Test positivity** | **Number tested** | **Proportion of number tested*** | **Proportion of population**** |
| WC | 15-24 | F | 16.8% | 46379 | 0.085 | 0.122 | 17.5% | 2308 | 0.047 | 0.033 | 21.2% | 13780 | 0.07 | 0.117 |
| WC | 15-24 | M | 17.7% | 55476 | 0.102 | 0.125 | 17.4% | 501 | 0.01 | 0.007 | 21.3% | 5287 | 0.027 | 0.018 |
| WC | 25-34 | F | 14.7% | 49315 | 0.091 | 0.109 | 17.5% | 11490 | 0.234 | 0.309 | 21.1% | 42011 | 0.214 | 0.335 |
| WC | 25-34 | M | 18.7% | 75497 | 0.139 | 0.127 | 19.0% | 5264 | 0.107 | 0.096 | 24.9% | 29409 | 0.15 | 0.133 |
| WC | 35-44 | F | 13.1% | 41970 | 0.077 | 0.088 | 14.9% | 9217 | 0.188 | 0.236 | 18.9% | 30768 | 0.157 | 0.165 |
| WC | 35-44 | M | 19.5% | 57221 | 0.105 | 0.092 | 18.4% | 8305 | 0.169 | 0.141 | 24.5% | 32769 | 0.167 | 0.117 |
| WC | 45-54 | F | 11.4% | 47338 | 0.087 | 0.08 | 12.6% | 4170 | 0.085 | 0.08 | 16.5% | 14294 | 0.073 | 0.049 |
| WC | 45-54 | M | 17.8% | 55017 | 0.101 | 0.071 | 15.9% | 4534 | 0.092 | 0.062 | 21.8% | 16265 | 0.083 | 0.044 |
| WC | 55-64 | F | 9.3% | 32476 | 0.06 | 0.055 | 9.8% | 1326 | 0.027 | 0.017 | 14.2% | 4351 | 0.022 | 0.009 |
| WC | 55-64 | M | 14.5% | 37046 | 0.068 | 0.046 | 13.3% | 1440 | 0.029 | 0.016 | 18.3% | 4985 | 0.025 | 0.01 |
| WC | >65 | F | 8.1% | 23527 | 0.043 | 0.05 | 10.2% | 246 | 0.005 | 0.002 | 15.5% | 931 | 0.005 | 0.001 |
| WC | >65 | M | 10.6% | 22295 | 0.041 | 0.036 | 9.5% | 275 | 0.006 | 0.003 | 16.7% | 1116 | 0.006 | 0.002 |

*Numerator (number in subpopulation who received a test) and denominator (total number in population in the cohort) taken from NHLS data

** Numerator (number estimated in subpopulation) and denominator (total estimated population) taken from Thembisa 2012 estimates

# Table A19 Tuberculosis (TB) testing measures amongst individuals in the NHLS data stratified by laboratory confirmed HIV and viral suppression statuses, excluding data from 2012.

|  | People not living with HIV | PLHIV virally Suppressed | PLHIV not virally suppressed |
| --- | --- | --- | --- |
| Number of TB testing periods | 6,810,645 | 1,218,558 | 4,444,202 |
| Number of diagnostic TB testing periods | 6,110,946 | 919,286 | 3,646,455 |
| TB testing periods per capita* | 0.03 | 0.09 | 0.30 |
| TB diagnostic testing periods per capita* | 0.03 | 0.07 | 0.25 |
| TB testing periods per TB individual** | 2.46 | 3.21 | 3.05 |
| TB testing periods per TB episode** | 2.35 | 3.03 | 2.86 |
| TB test positivity** | 9.03% | 9.93% | 13.07% |
| Diagnostic TB test positivity** | 7.61% | 7.64% | 11.69% |
| Number of positive TB testing periods | 614,690 | 121,005 | 580,731 |
| Number of TB episodes | 495,219 | 121,913 | 428,559 |
| Number of people with laboratory diagnosed TB | 473,311 | 115,197 | 402,078 |
| Number of people with laboratory diagnosed TB per capita* | 0.0020 | 0.0089 | 0.0273 |
| Mean number of unique diagnostic test types*** | 1.9100 | 2.3400 | 2.0400 |

*Denominators from Thembisa estimates

**Denominators from NHLS data

***Individuals could receive a maximum of 7 unique diagnostic tests: culture, DST1, DSTX, Xpert, LPA, PCR, or smear
